# *Copaifera langsdorffii* Novel Putative Long Non-Coding RNAs: Interspecies Conservation Analysis in Adaptive Response to Different Biomes

**DOI:** 10.3390/ncrna4040027

**Published:** 2018-10-08

**Authors:** Monica F. Danilevicz, Kanhu C. Moharana, Thiago M. Venancio, Luciana O. Franco, Sérgio R. S. Cardoso, Mônica Cardoso, Flávia Thiebaut, Adriana S. Hemerly, Francisco Prosdocimi, Paulo C. G. Ferreira

**Affiliations:** 1 Laboratório de Biologia Molecular de Plantas, Instituto de Bioquímica Médica Leopoldo de Meis, Universidade Federal do Rio de Janeiro, Rio de Janeiro 21941-599, Brazil; monica.danilevicz@gmail.com (M.F.D.); flaviabqi@gmail.com (F.T.); hemerly@bioqmed.ufrj.br (A.S.H.); 2 Laboratório de Química e Função de Proteínas e Peptídeos, Centro de Biociências e Biotecnologia, Universidade Estadual do Norte Fluminense Darcy Ribeiro, Rio de Janeiro 28013-602, Brazil; kcm.eid@gmail.com (K.C.M.); thiago.venancio@gmail.com (T.M.V.); 3 Instituto de Pesquisas Jardim Botânico do Rio de Janeiro, Diretoria de Pesquisa Científica, Rio de Janeiro 22460-030, Brazil; lfranco@jbrj.gov.br (L.O.F.); sergio@jbrj.gov.br (S.R.S.C.); mcardoso@jbrj.gov.br (M.C.); 4 Laboratório de Genômica e Biodiversidade, Instituto de Bioquímica Médica Leopoldo de Meis, Universidade Federal do Rio de Janeiro, Rio de Janeiro 21941-599, Brazil; prosdocimi@bioqmed.ufrj.br

**Keywords:** novel lncRNA, lncRNA conservation, *Copaifera*, epigenetics, adaptive response

## Abstract

Long non-coding RNAs (lncRNAs) are involved in multiple regulatory pathways and its versatile form of action has disclosed a new layer in gene regulation. LncRNAs have their expression levels modulated during plant development, and in response to stresses with tissue-specific functions. In this study, we analyzed lncRNA from leaf samples collected from the legume *Copaifera langsdorffii* Desf. (copaíba) present in two divergent ecosystems: Cerrado (CER; Ecological Station of Botanical Garden in Brasília, Brazil) and Atlantic Rain Forest (ARF; Rio de Janeiro, Brazil). We identified 8020 novel lncRNAs, and they were compared to seven Fabaceae genomes and transcriptomes, to which 1747 and 2194 copaíba lncRNAs were mapped, respectively, to at least one species. The secondary structures of the lncRNAs that were conserved and differentially expressed between the populations were predicted using in silico methods. A few selected lncRNA were confirmed by RT-qPCR in the samples from both biomes; Additionally, the analysis of the lncRNA sequences predicted that some might act as microRNA (miRNA) targets or decoys. The emerging studies involving lncRNAs function and conservation have shown their involvement in several types of biotic and abiotic stresses. Thus, the conservation of lncRNAs among Fabaceae species considering their rapid turnover, suggests they are likely to have been under functional conservation pressure. Our results indicate the potential involvement of lncRNAs in the adaptation of *C*. *langsdorffii* in two different biomes.

## 1. Introduction

*Copaifera* is a genus of native trees from Latin American tropical regions and Western Africa. The species *Copaifera langsdorffii*, *Copaifera reticulata*, *Copaifera cearensis*, *Copaifera multijuga* among others are popularly known as “copaíba” [1,2]. The *Copaifera* sp. oil is extracted through V-shaped cut in the stem bark and has been used by indigenous populations from the Brazilian Amazon as a powerful antimicrobial, anti-inflammatory and for “overall healing purposes” [3]. Many of these alleged features have been investigated by pharmacological studies, confirming its anti-inflammatory capacity [3,4,5] and proving it to be an efficient alternative to treat dental infections [6], gastrointestinal disorders [7], endometriosis [8], skin ulcers [9,10], and to be applied to skin scaffold implants increasing tissue angiogenesis [11]. The copaíba oil resin has been shown to exert larvicidal activity against *Aedes aegypti* [12,13], and antibacterial activity in vitro [6,14,15,16]. The leaf extracts also presented leishmanicidal and antimalarial activities [17,18,19,20,21], also being effective as a biopesticide against lepidoptera [22].

The amount of copaíba oil production is influenced by the climate and soil conditions, as more oil is produced in locations with clay soil, during the wet season [23]. In Brazil, *C*. *langsdorffii* Desf. is a widely occurring species, included in a broad range of ecosystems like Cerrado, Atlantic Rain Forest and Caatinga, which are very distinct biomes [23,24], requiring diverse adaptive mechanisms. Such plasticity to adapt to different biomes is a complex regulation, involving several genetic, evolutionary and epigenetic fine tuning, which may also include long non-coding RNAs (lncRNAs). 

lncRNAs are considered to be RNA transcripts, defined as longer than 200 bp, with no apparent protein coding capacity [25,26,27,28,29,30]. They hold many resemblances with messenger RNA (mRNA), such as similar epigenetic marks to promote expression and binding sites for RNA polymerase II at their genome loci. They are often polyadenylated at 3′ end and receive 5′ CAPs, especially when acting outside the nucleus [25,26,27,30,31]. Because they are (i) less conserved than mRNA at the sequence level across different species and (ii) regularly transcribed at low levels, lncRNAs were once considered transcriptional noise. lncRNAs tends to form secondary and tertiary structures, in which the molecule conformation is crucial to regulate their targets, leading to their functional domains and genomic positions to be more conserved than their sequence [32].

lncRNAs interspecies conservation may be influenced by the regulatory mechanism they play, whereas their sequence conservation depends on whether (a) the lncRNA molecule acts as a regulator or (b) the simple transcription of the lncRNA regulates its target gene [25]. Most functional lncRNAs undergo post-transcriptional processing and retain higher conservation of splice sites. This indicates that they most likely function in the mature form [25,30]. In vertebrates, it is argued that transposable elements and bidirectional transcription may play an important role in the evolution and rapid turnover of lncRNAs [26,29].

There are several types of lncRNAs classified according to their genomic positions such as: Sense, natural antisense, bidirectional, intronic and intergenic; and they may also act through cis or trans regulation [28,31,33]. lncRNAs exhibit relatively low expression patterns compared to mRNAs, showing a specific profile depending on the (i) tissue or cell type observed [34,35], (ii) developmental stage [36,37,38] and (iii) environmental stress response [37,39,40,41] 

A considerable amount of lncRNAs may act as chromatin regulators [42,43,44,45]. For example, *APOLO*, a lncRNA responsive to auxin, interacts with the chromatin, leading to a loop formation encompassing the *PID* gene (key regulator of polar auxin transport) and regulating its expression [46]. *HOTAIR*, *ANRIL* and *KCNQ1OT1* are also known to bind to more than one histone-modifying complex, acting as regulators [47,48]. *COLDAIR* [49], *COOLAIR* [50] and *COLDWRAP* [51] are lncRNA described to act regulating the FLOWERING LOCUS C (FLC), associated with the Polycomb protein complex to stably repress FLC during the vernalization process, prompting adaptive fitness and development in *Arabidopsis thaliana*. In rice, it was shown that photoperiodic-sensitive male sterility (PSMS) is carried out by a lncRNA called *LDMAR*, in which a single-nucleotide polymorphism (SNP) led to a change in its secondary structure and subsequently the repression of its expression at long day conditions [52]. Several other lncRNA have their functions experimentally tested, such as *HDI* promoting photomorphogenesis in red light [53], and *NERDL* association to wood formation in *Populus tormentosa* [54].

In order to modulate the plants response to adaptive stress, such as inorganic phosphorus starvation, the lncRNA *cis-NAT PHO1*;*2* acts regulating phosphate (Pi) homeostasis as a translational enhancer of *OsPHO1*;*2*, increasing its uptake when over-expressed in a mutated rice lineage [55]. Evidence of lncRNA involved in stress response and adaptation was also observed in nitrogen deficient *Populus* [40], maize under drought and nutrient stress [56,57] and *Arabidopsis thaliana* submitted to salt stress [58]. Moreover, lncRNAs have also been found to act as small RNA (sRNA) precursors [27,59] or acting as bait to sRNA regulation by target mimicry [60,61,62]. Collectively, lncRNAs have been implicated in various cell and molecular processes, including post-transcriptional regulation, post-translational regulation of protein activity and protein re-localization, organization of protein complexes, cell–cell signaling and intrinsically connected to adaptive fitness and overall plant processes [28,29,31,33].

In this study, we identified 8.020 novel lncRNAs through bioinformatics analysis, of which approximately 565 were shown to be up-regulated above 5 times in Cerrado (CER) and Atlantic Rain Forest (ARF) populations, which might be related to their adaptation to such diverse environments. lncRNAs tend to have their primary sequence evolving under a relaxed constraint, being less likely to present high interspecies sequence similarity. Nevertheless, through the comparison to other Fabaceae genomes, there were 1747 putative lncRNA conserved, some of them presenting different expression profiles among the plant populations. Additionally, we aligned the copaíba transcripts to same family species transcriptomes and found 2194 aligned to at least one species transcriptome. There were 1141 lncRNAs which overlapped, being aligned in both analyses, from which some of them had differential expression. Also, we were able to identify one single transcript that matched a known *Glycine max* lncRNA [63]. Amongst the conserved lncRNAs with higher degrees of differential expression that had their secondary structure stability predicted, 186 transcripts are predicted to be stable. The emerging studies involving lncRNAs function and conservation have shown their involvement to several types of biotic and abiotic stress. Thus, the conservation of lncRNAs among Fabaceae species considering their rapid turnover, suggests they are likely to have been under functional conservation pressure [32,64,65]. The differential expression observed for hundreds of lncRNAs suggests that they take part in regulatory pathways that lead to adaptive responses in copaíba, which has high health and economic interest.

## 2. Results

### 2.1. Identification of Novel and Differentially Expressed lncRNA

**Figure 1:**
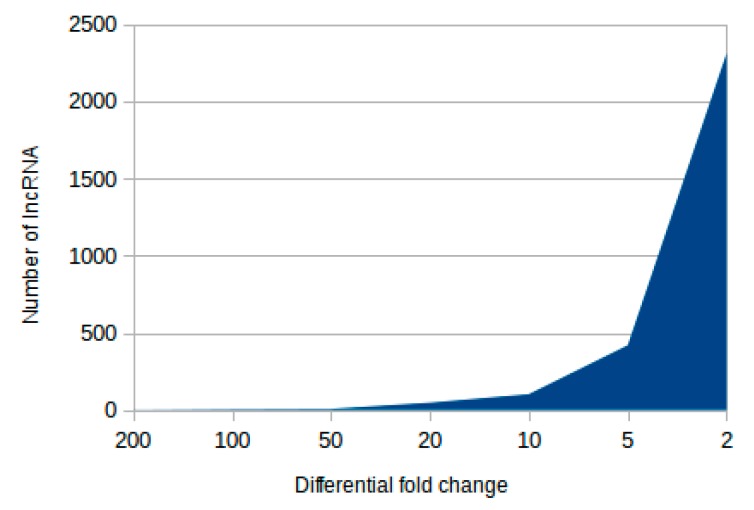
Putative lncRNAs identified in CER and ARF samples and its fold change regulation in comparison to each other: There were 2893 differentially regulated transcripts identified from a total of 8020 copaíba lncRNAs. The majority of the transcripts were 2 to 5 times differently expressed on either sample, yet there were 565 transcripts regulated above 5-fold on either sample.

In order to identify lncRNAs and elucidate their adaptive roles, we sequenced the transcriptomes of *C*. *langsdorffii* leaves obtained from trees growing in two different ecosystems, the (i) Atlantic Rain Forest (ARF, humid condition) and the (ii) Cerrado (CER, dry forest) in Brazil during the drought season for both locations. Approximately 75 million high quality reads from each library were used for *de novo* assembly, and there was a total of 138,175 and 199,556 transcripts assembled from ARF and CER, respectively. A series of filters were applied to these transcripts to remove potentially coding transcripts, such as coding potential calculator (CPC) (v0.9-r2) [66] and predictor of long non-coding RNAs and messenger RNAs based on an improved k-mer scheme (PLEK) (v1.2) [67], and only transcripts classified as non-coding by both software were compared to establish one-to-one correspondence between CER and ARF. Reads were remapped against the assembled transcriptomes with Bowtie2 [68] and transcriptional levels estimated with Cufflinks v2.2.1 [69] (Table 1). Transcripts with at least 1 RPKM (reads per kilobase of transcript per million mapped reads) were kept for downstream analyses (8020 transcripts) (Table S1). The majority of these (2312 transcripts) presented over 2-fold regulation, while 565 transcripts were regulated above 5-fold (Figure 1, Table S2).

### 2.2. Interspecies lncRNA Conservation Analysis

#### 2.2.1. Positional Conservation and Genome Alignment Analysis

**Figure 2:**
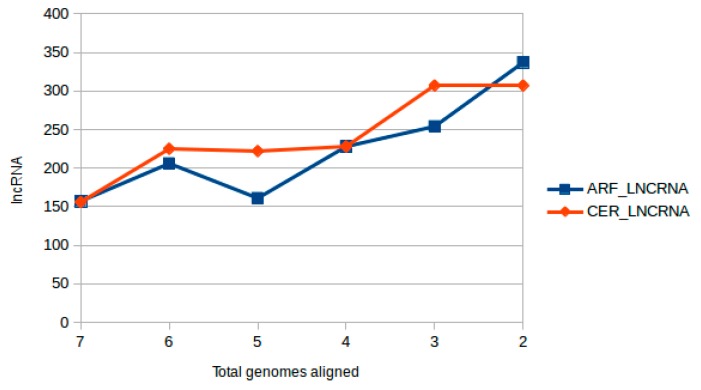
lncRNA interspecies conservation and genome alignment analysis: the graph shows the total number of genomes versus the total number of transcripts aligned to Fabaceae genomes (*Vicia faba*, *Glycine max*, *Medicago truncatula*, *Phaseolus vulgaris*, *Lotus japonica*, *Vigna unguiculata* and *Cicer reticulatum*). The blue line represents the lncRNA gathered from the ARF samples and the red line represents the lncRNA gathered from the CER sample, their alignment profile is very similar as expected, around 1800 transcripts aligned to at least one of the genomes used.

Since there is no reference genome for *C*. *langsdorffii*, we performed a similarity search using Bowtie2 (v2.3.4.1) [68] against the genomes of the following related species: (i) *Vicia faba*, (ii) *Glycine max* [70], (iii) *Medicago truncatula* [71], (iv) *Phaseolus vulgaris* [72], (v) *Lotus japonica* [73], (vi) *Vigna unguiculata* [74] and (vii) *Cicer reticulatum* [75]. From the alignments, we found 1747 and 1879 transcripts respectively from ARF and CER samples to match at least one of the genomes used. From which we noticed 156 transcripts aligned to all seven Fabaceae genomes in both CER and ARF samples (Figure 2), then being considered highly sequence conserved lncRNA transcripts [29,64,65].

**Figure 3:**
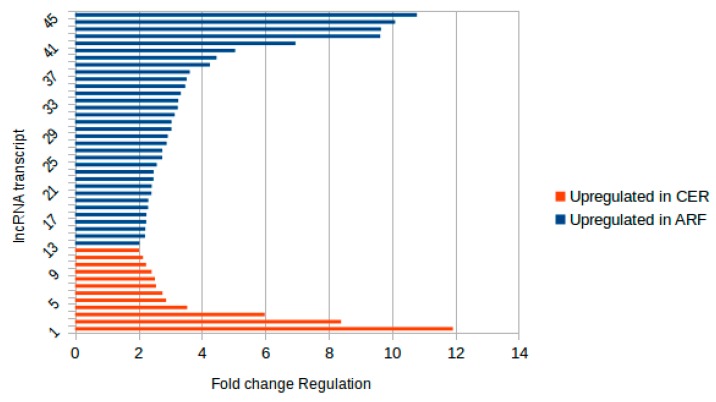
Subset of transcripts which aligned to all Fabaceae genomes presenting differential expression: From 156 transcripts which aligned to all genomes analyzed, there were 45 copaíba lncRNAs upregulated in either condition represented in this graph. Each transcript is represented by a single bar. In red are indicated the lncRNAs upregulated in CER samples in relation to ARF. In blue are indicated the lncRNAs upregulated in ARF samples in relation to CER. The x axis indicates the fold change regulation of the transcripts.

From the subset of 156 copaíba lncRNAs aligned to all seven Fabaceae genomes, 45 transcripts presented above 1-fold differential regulation (Figure 3). To understand if the copaíba lncRNA transcripts aligned to all seven Fabaceae genomes hold positional conservation, we selected 10 transcripts exhibiting at least 5-fold up-regulation to retrieve the information regarding their genomic locations. For this analysis, we used the reference genomes of *P*. *vulgaris*, *G*. *max* and *M*. *truncatula*, as they are better assembled and annotated. However, the genes closely located to the lncRNA loci are described only as “hypothetical protein-coding”, or “plant-like protein”. Furthermore, none of the 6133 lncRNA annotated in the *G*. *max* genome were located close to copaíba putative lncRNA alignment locations. The *M*. *truncatula* or *P*. *vulgaris* annotation files had no information on lncRNAs.

#### 2.2.2. Identification of Putative *C*. *langsdorffii* lncRNAs in EST Sequences of Other Fabaceae Species

We downloaded expressed sequence tag (EST) and complementary DNA (cDNA) libraries from Phytozome and NCBI databases from six Fabaceae species to identify whether the copaíba lncRNA transcripts that aligned to the genomes could also be found in available transcriptome libraries, which comprise both mRNA and poly-A non-coding RNA (ncRNA). lncRNAs have a specific expression profile, considered to be lower than mRNAs. The likelihood of expression is highly influenced by tissue and specific condition [25,37,39]; therefore, the identification of copaíba lncRNA in Fabaceae transcriptome is susceptible to being underestimated. This is due to the fact that transcripts specific profile of expression and relatively low amounts may not be observed, even if present in the reference transcriptomes. 

**Figure 4:**
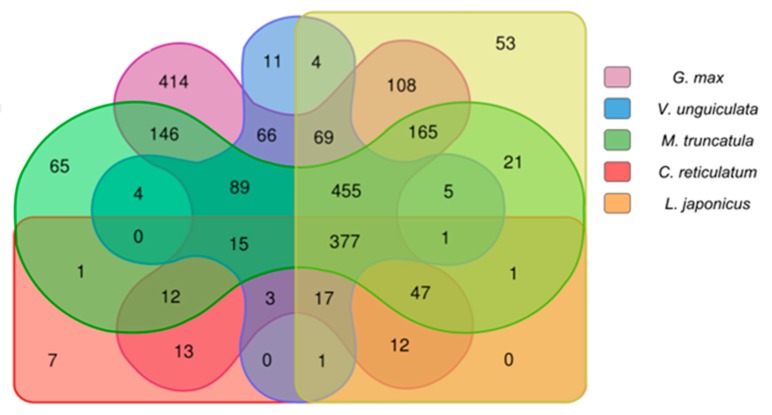
Count of copaíba lncRNAs aligned to each Fabaceae transcriptome with BLASTN (50% identity): This diagram shows the amount of copaíba lncRNA aligned to the transcriptome of each species, segregated by a color pattern indicated on the legend. In the colored overlapped area are the transcripts which were aligned to more than one species transcriptome, and its respective amount. There are only five species shown in this diagram, for illustration purposes we left out *P*. *vulgaris*.

**Figure 5:**
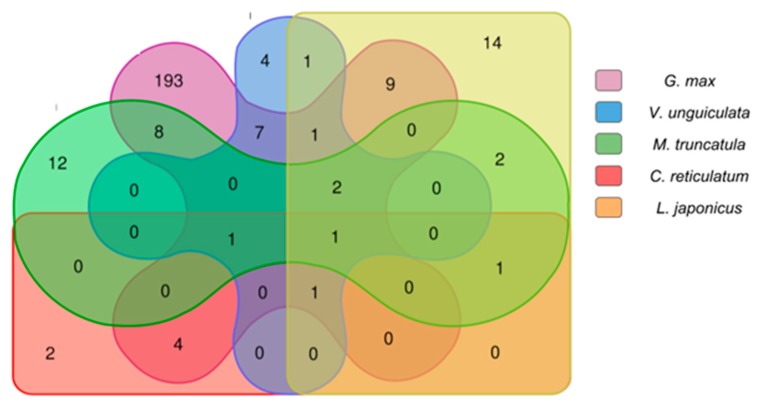
Count of copaíba lncRNAs aligned to each Fabaceae transcriptome with BLASTN (90% identity): This diagram shows the amount of copaíba lncRNA aligned to the transcriptome of each species, segregated by a color pattern indicated on the legend. In the colored overlapped area are the transcripts which were aligned to more than one species transcriptome, and its respective amount. There are only five species shown in this diagram, for illustration purposes we left out *P*. *vulgaris*.

We used Basic Local Alignment Search Tool (BLASTN) (v2.2.31+) [76] to search putative copaíba lncRNAs against downloaded transcript sequences. Two identity thresholds, 50% and 90%, were used in this analysis, along with e-value and coverage threshold of 10^−15^ and 50%, respectively. We found that 27% (2194 lncRNA) aligned to at least one species’ transcriptome (using 50% identity filter on BLASTN) (Figure 4). While only 3.3% (264 lncRNA) aligned with 90% identity to at least one Fabaceae expressed transcripts library and a single transcript (JCF44_0000056614) aligned to five species. It is remarkable to notice that 227 transcripts aligned to *G*. *max* with more than 90% identity, which might be due to *G*. *max* to being more intensively studied species than the others (Figure 5).

#### 2.2.3. Comparison Analysis of Conserved Putative lncRNAs

**Figure 6:**
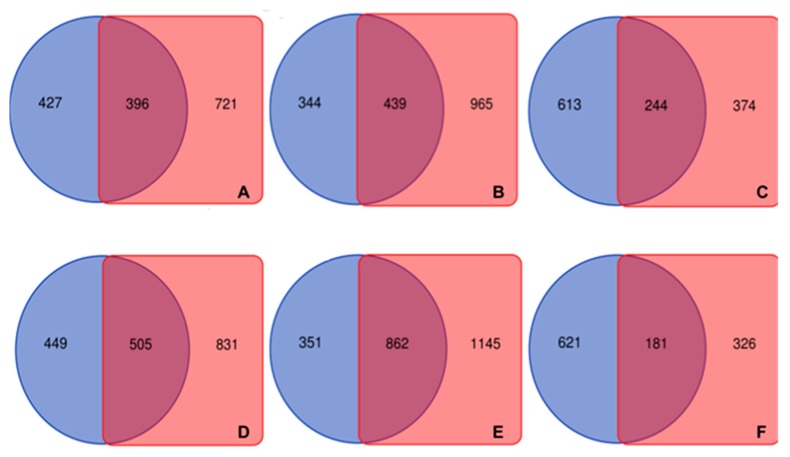
BLASTN comparison of copaíba lncRNA aligned to each species genome and complementary DNA (cDNA): In the venn diagram is compared the amount of copaíba transcripts aligned to the genome (blue circle), to the transcriptome (red square) or to both (overlapped area). (**A**) is the *V*. *unguiculata* comparison, (**B**) is *M*. *truncatula*, (**C**) is *P*. *vulgaris*, (**D**) is *L*. *japonicus*, (**E**) is *G*. *max* and (**F**) is *C*. *reticulatum*. It is possible to observe that G. max, *L japonicus* and *M*. *truncatula* presented a higher number of overall aligned transcripts and also overlapped ones.

**Figure 7:**
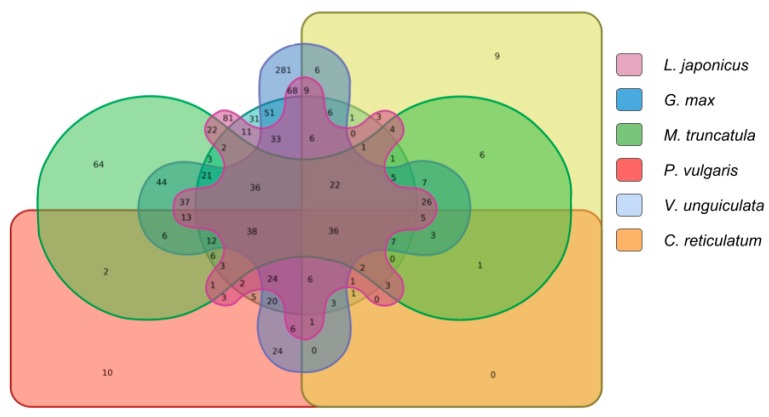
Total transcripts aligned to Fabaceae genomes and transcriptome: This diagram shows the amount of copaíba lncRNA aligned to the both genome and transcriptome of each species segregated by a color pattern indicated in the legend. In the colored overlapped area are the transcripts which were aligned to more than one species genome and transcriptome, and its respective amount.

To assess whether the copaíba lncRNAs aligned to Fabaceae transcribed libraries were also aligned to Fabaceae genomes, we used the Bowtie2 [68] aforementioned result, aiming to determine the number of transcripts that are present in the genome and that also are actively transcribed in the cDNA, EST libraries used (Figure 6, Table S3). Thus, we identified 1141 transcripts that aligned to both genome and transcriptome comparisons of at least one species. From this total, there were 36 copaíba lncRNA transcripts which aligned to both genome and transcriptome of six Fabaceae species (Figure 7).

#### 2.2.4. Expression Analysis of the Conserved Putative lncRNAs among Fabaceae

**Figure 8:**
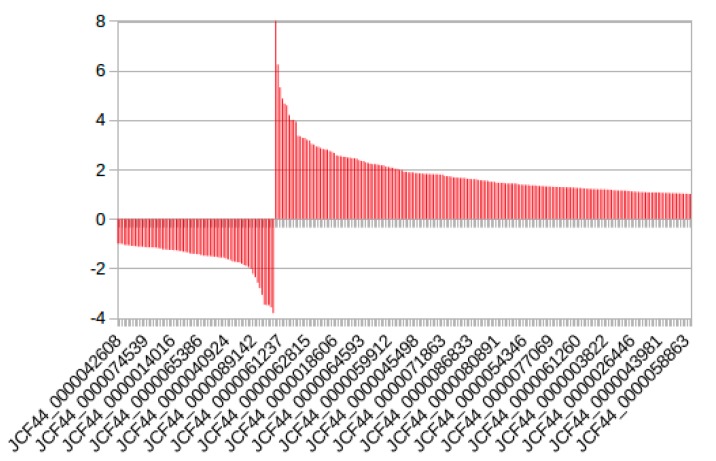
log2fold change comparison of copaíba lncRNA conserved in Fabaceae species: A total of 1141 lncRNA aligned to multiple Fabaceae genomes and transcriptomes. Comparing the expression of the ARF against CER samples, we found 256 transcripts that presented log2fc above 1. The graph displays the regulated transcripts of ARF samples compared to CER, each bar corresponds to a single transcript. It is possible to notice that 24 transcripts are regulated above 3-fold.

Most of the lncRNA transcripts that aligned to both genome and transcriptomes of Fabaceae species displayed similar RPKM values for CER and ARF. However, several lncRNAs (256 out of 1141) were regulated at either condition above 2-fold, indicating that they might be involved with the adaptation to different environments (Figure 8). From the total transcripts which aligned to both genome and transcriptome of Fabaceae species, it is possible to identify the formation of two clear groups of putative lncRNAs formed in each condition. The values and identification of the putative lncRNA are available at Table S4.

#### 2.2.5. Identification of Known lncRNA

In order to investigate conserved lncRNAs in closely related species, we used the putative transcripts obtained and compared with BLASTN against CANTATAdb 2.0 and GREEnc, which are lncRNA databases for plants. The lncRNAs of *S*. *bicolor*, *G*. *max*, *M*. *truncatula* and *P*. *vulgaris*, summed up approximately 32,000 sequences. This comparison found a single transcript (JCF45_0000011974/JCF44_0000015840) that matched a *G*. *max* lncRNA from CANTATA database (CNT2032069) with 90% identity and e-value of 4 × 10^−97^. This single transcript is 6.5 fold up-regulated in ARF samples. This transcript was also conserved in *G*. *max*, *L*. *japonicus* and *P*. *vulgaris* genome analysis.

The high sequence identity of the JCF45_0000011974/JCF44_0000015840 transcript with other legume genomes and with a *G*. *max* lncRNA transcript indicates that it might play biological roles, which could be related to the adaptation to different niches, as it is differentially regulated between CER and ARF populations.

### 2.3. Stem-Loop Secondary Structure of Regulated Putative lncRNA

Recent studies have suggested that the secondary and even tertiary structures of lncRNAs are conserved and critical for the transcript to be functionally active [77,78]. The secondary structure of lncRNA is regarded as one of the multidimensional conservation pressures that long non-coding transcripts can suffer [79] and as a result of their sequence length, most RNA transcripts are prone to form secondary structure [64]. However, ncRNA present some distinguishable features, such as higher thermostability than coding transcripts, also (ii) their temperature melting (Tm) is significantly higher and (ii) they have greater negative free energy values (minimum free energy—MFE) [79,80]. Another study observed that functional transcripts tend to present higher in silico second structure stability (with greater negative MFE), suggesting a link between secondary structure stability and functionality [81,82].

There are lncRNAs in which the secondary structure dictates their functionality, in humans the lncRNA MEG3 acts as a tumor suppressor based in the structure rather than in the primary sequence conservation [83], also the steroid receptor RNA activator (SRA), one of the few lncRNA that has its secondary structure experimentally defined, is reported to interact with many proteins and be related to breast cancer development. Although SRA primary sequence is mutated, the secondary structure, and the nucleotides involved in the stabilization of the structure are highly conserved, suggesting their direct involvement in the lncRNA functionality [84]. Experimental analysis comparing the folding energy of lncRNAs and mRNAs were capable of differentiating lncRNA based on their higher MFE [79].

**Figure 9:**
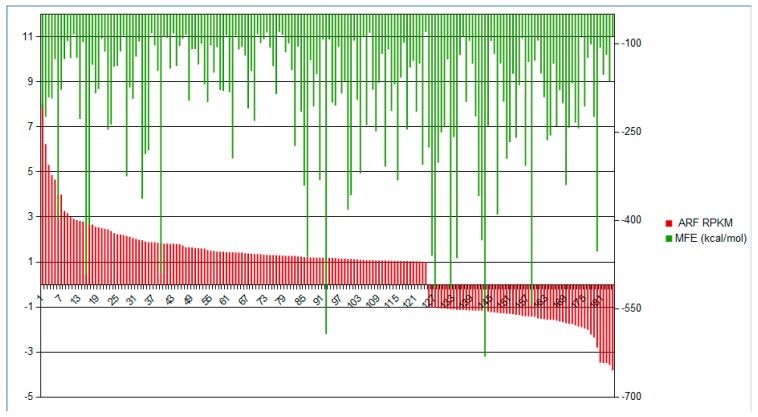
Predicted MFE value and lncRNA relative expression RPKM: The regulated 186 conserved lncRNAs, which were folded to predict their secondary structure stability and presented MFE below −80 kcal/mol. The red bars represent the expression regulation (log2FC) of each lncRNA from ARF samples in comparison to CER. The green bars are the transcripts respective MFE value.

Therefore, we performed folding analysis using ViennaRNA (v2.4.8) [85]. In which the stability of secondary structure of RNA can be inferred by MFE, regarding any values below −80 kcal/mol to be structurally stable [77,84]. We selected 256 lncRNAs presenting log2FC above 1 from the set of 1141 conserved lncRNAs in both Fabaceae genome and transcriptome analysis. The majority of lncRNAs (186) presented MFE below −80 (Figure 9). Once these structures were shown to be stable, it is expected that they may have a functional role in which a secondary structure formation is likely to be meaningful [77,79]. At the Table S5, the lncRNA identification, RPKM and predicted MFE values are available. 

### 2.4. RT-qPCR Analysis of Copaíba lncRNA Expression

To validate the expression and sequence of the lncRNA proposed by the RNA-Seq, we selected six conserved lncRNA, considering their high conservation (genome and transcriptome analysis), and regulated expression between the two populations, together with the low MFE value for the predicted structure. The reverse transcription real-time polymerase chain reaction (RT-qPCR) analysis of the lncRNA showed validates the RNA-Seq indication, the transcripts were differentially regulated between the conditions. Since the RNA-Seq was performed from a pool of 10 individuals for each sample, an oscillation was expected regarding the precise differential expression (Figure S1).

### 2.5. Computational Identification of miRNA and lncRNA Interactions

lncRNA transcripts have been previously reported to interact with microRNA (miRNA), acting as their targets or as a decoy for them [58,59,60,61,62]. In plants, there are tools dedicated to the identification of such interactions, like psRobot [86] and psMimic [61]. Analyzing the 1141 conserved copaíba lncRNA transcripts against Fabaceae mature miRNA sequences [87], we identified 94 lncRNA-miRNA interactions with 76 uniquely aligned miRNA. Some of the miRNA families are predicted to bind to more than one lncRNA targets, some of which were regulated in the RNA-Seq, as indicated in Table S6. The lncRNA may also act as endogenous target mimicry (eTM), a novel regulatory pathway in which the non-coding transcript acts as decoy for a miRNA, preventing it from binding to its target, leading to an increased expression of its target mRNA [88,89,90,91]. Then, to predict the potential interaction between conserved copaíba lncRNA and the known Fabaceae miRNA, we performed a target mimicry analysis through psMimic [61] In this analysis, 32 lncRNAs transcripts are indicated to potentially act as decoy targets for 31 highly conserved miRNA families which are generally involved in plants’ stress response [90,92,93,94], such as: miR408, miR156, miR164, miR169, miR4392, miR395, miR2673, miR2638, miR7696 and miR11073 (the detailed information is indicated in Table S7).

## 3. Discussion

Forest trees are a unique group to study adaptability traits, based on their life span and endurance to biotic and abiotic stress [95]. Thus, in the present study we identified 8020 putative lncRNAs, some of which regulated above 2- and 5-fold in either copaíba population originated from different biomes. Additionally, a comprehensive analysis of copaíba coding transcripts is being conducted by our group (Franco et al., in preparation), leading to a deeper understanding about their adaptability to these different environments at mRNA and epigenetics level, which is critical to copaíba management and conservation [95,96]. Similarly, Xu et al. [97] performed a study with *Miscanthus lutarioriparius* populations from two different environments and observed an expression profile in which the lncRNA presented higher fold change expression than usually observed with mRNA, suggesting them to be more sensitive and responsive to environmental changes than coding transcripts.

Positional conservation in the genome analysis of lncRNA loci neighboring specific orthologous genes plays an interesting role, particularly when their primary and secondary structures are not completely conserved through species. For example, in humans, a small segment of *AIRN* lncRNA overlaps with the *IGF2R* promoter (insulin-like growth factor 2 receptor) and is sufficient to cause *IGF2R* silencing. This phenomenon was also identified in other lncRNAs [32,34]. Through previous interspecies comparison [29] it was observed that the positional conservation may act by regulating the target gene solely by the transcription of the lncRNA.

In the present copaíba analyses, a total of 22% of the lncRNAs aligned to at least one of the genomes analyzed. The observed amount of lncRNA conservation is coherent with the literature, since the conservation of lncRNA among the same family is much smaller than that of protein-coding genes [25,29,64]. A conservation analysis between human and other placental mammals estimates only 44% of the lncRNA is conserved, while their promoter also seems to be under conservative pressure [98]. In plants, between *Z*. *mays* and *S*. *bicolor* there are approximately 25% lncRNA conserved, while *Z*. *mays* compared to *A*. *thaliana* presented only 2% conservation [99]. Thus *C*. *langsdorffii’s* 22% conservation along the Fabaceae family lies in the expected conservation threshold. Within these conserved putative lncRNAs, some were regulated in either condition and it is possible to observe that the majority of them are up-regulated in ARF plants, with a high fold change. The high conservation and regulation among these transcripts suggest that they are under evolutionary constraints, possibly involved in the plant regulatory machinery [32].

Often the lncRNA primary sequence can be degenerated while the position is maintained, indicating that the transcription itself is enough to function as an epigenetic regulator to closely related genes [25,32,64,80,97]. Wu et al. [89] identified an intronic lncRNA, which binds to Curly leaf (CLF) acting as a co-repressor of *AGAMOUS (AG)*, and it also encodes four new ncRNAs. A study performed comparing putative lncRNA among the *Brassicaceae*, *Aethionemeae* and *Cleomaceae* families uncovered several transcripts that were thought to be lineage-specific, instead they were in fact positionally conserved, although sequence divergent [32]. Moreover, comparing the copaíba lncRNA to several Fabaceae genomes has many advantages, enabling the identification of the transcripts in other species even when they are not being actively transcribed at a given condition. Mapping the lncRNA transcripts to other species cDNA and EST sequences is a way to confirm that it is actively transcribed, and potentially functional [25,26]. Some previous studies that performed this analysis using vertebrates’ lncRNA noticed that some transcripts may hold sequence similarities to genomic untranscribed regions [29]. In our study, a higher percentage of lncRNA was paired with EST and cDNA libraries than to the genomic alignment analysis; it might be due to lncRNA processing, which prevents the lncRNA from being correctly mapped to the genome or to the stringent parameters used. The transcripts alignment analysis suggests more orthologous lncRNAs are being actively transcribed throughout the Fabaceae family than initially estimated by genomic analysis.

Regarding that lncRNAs frequently present low expression profile and tendency to be expressed in a tissue or condition specific manner [25,64,80], the transcripts that didn’t align to any of the expressed Fabaceae libraries analyzed may still be conserved, although not expressed in the particular condition in which the library samples were taken from. It can be illustrated by the fact that, when comparing copaíba lncRNAs to two databases of putative plants lncRNAs, there was a single transcript that aligned to a known *G*. *max* lncRNA and mapped to three Fabaceae genomes but didn’t align to *G*. *max* EST and cDNA libraries. Hence, it is essential to understand if the comparison analysis is insufficient to state whether the transcript is expressed or not in a given species.

Moreover, we observed that 14.2% of copaíba lncRNA overlapped the genomic and transcription comparative analysis, while the majority presented similar expression levels in both CER and ARF samples, there were 256 transcripts regulated in either sample above 2-fold. In Figure 8, we can detect a tendency to form two distinct expression profiles among the samples, in which 17 lncRNAs were strongly up-regulated. Considering the samples are from the same type of tissue, and similar developmental stages, it is reasonable to infer that if the lncRNAs identified are functional, the RPKM differential expression between the samples is associated to the plants’ response to environmental stimuli. It should be mentioned however that, although there are no replicates in the experiment, RNA samples were prepared from a pool of 10 plants from each local. 

During the in silico folding prediction of 256 most regulated lncRNAs, which were conserved at genome and transcriptome level, most of the transcripts were regarded to have a stable secondary structure. Thus, a further investigation of these transcripts regarding their functionality is needed, since their structural stability and post-transcriptional processing indicates they are likely to regulate their target expression as molecule, not solely by being transcribed. Thus, the functional characterization and possible targets identification are the natural research directions to further understand the role of lncRNAs in the adaptive response of copaíba and possibly other closely related woody plants. 

The miRNA–lncRNA interaction analysis predicted that several of the conserved transcripts might act as potential targets or decoys for miRNA. This interaction has already been observed in other studies [60,61]. The number of putative lncRNA–miRNA targets was similar to Nithin et al. [90] in which the interaction of those ncRNAs was aimed to the crops’ improvement. The majority of the miRNA predicted to interact with copaiba transcripts there is reported to be involved in the plants development and response to stress, for instance the miR408 that targets copper protein and plantacyanin genes [100], is responsive to several abiotic stresses [99,100,101,102,103,104,105]; the miR156 targets *SPL* transcription factor genes, which are strongly modulated in response to environmental changes [106,107,108,109]; the miR164 negatively regulates *NAC* transcription factors during stress [110,111,112,113,114]; also miR169 is extensively studied due to its involvement in the plants response to pathogens infections [94,115,116], and to increase crops resistance to environmental changes [93,117,118].

The identification of the copaiba lncRNAs present an addition to the understanding of lncRNAs in tree plants; several of the transcripts identified are also conserved in other Fabaceae species, which as we understand is the first lncRNA conservational study to analyze this group comparing the genomic and expression data of this plant family. The miRNA–lncRNA predicted interaction presents and interesting cue to the possible role of those lncRNAs in copaiba, however a deeper analysis is necessary to functionally characterize the transcripts.

## 4. Materials and Methods

### 4.1. Plant Material Collection

The *Copaifera langsdorffii* leaf samples were collected from two different biomes, ten individual samples were taken from Atlantic Rain Forest (ARF), at Área de Proteção Ambiental da Bacia do Rio São João—Mico Leão, Silva Jardim, RJ, Brazil. And another ten individual samples were collected from Cerrado (CER) ecosystem at Estação Ecológica do Jardim Botânico de Brasília—EEJBB, Distrito Federal, Brazil, in August, during the conspicuous annual dry season. The collected plants specimen was deposited at the Botanical Garden of Rio de Janeiro under the identification number of RB 773246 (CER) and RB 773299 (ARF). All biological material harvested for the expression analysis was placed in RNA later-like buffer, kept at −80 °C until extraction.

### 4.2. RNA Extraction to Sequencing

RNA extraction was performed individually from samples collected from Atlantic Rain Forest and Cerrado. The total RNA was extracted following the modified Japelaghi protocol [119], 10 µg of each RNA samples were sent to Fasteris Life Sciences SA (Plan-les-Ouates, Switzerland), where the quality and quantity was ascertained by Qubit and Bioanalyzer Nano Chip. Ten individual leaf samples from each ecosystem were selected according to their quality, after passing through poly-A selection protocol. The treated samples were polled together in equal concentration amounts for sequencing. The ARF and CER samples were named JCF45 and JCF44 respectively, in the. multiplex sequencing performed on Illumina HiSeq 2000I using the single-end 76 cycle protocol .

### 4.3. De Novo Transcriptome Assembly and Gene Expression Profiling

Sequencing reads were checked using FastQC and searched against NCBI nr database using BLASTN (megablast, e-value 1 × 10^−5^; alignment length ≥90 and identity ≥80%) [76]. Reads aligning to non-plant organisms were removed. Raw reads were pre-processed using Trimmomatic (v0.36) [120] for trimming adapters, trailing 15 bases and tailing 5 bases for each read and other quality cleaning. De novo transcriptome assembly was performed using Trinity (v2.3.2; default settings) [121]. Fasta headers were renamed according to the sample ids (e.g., JCF44_xxx and JCF45_xxx). One-to-one correspondence between the transcriptomes was detected by bi-directional BLASTN (v.2.2.31+) [76]. Stringent criteria were used for this analysis and only top hits with e-value 1 × 10^−10^, query coverage of ≥50%, identity ≥90%, bit-score≥50 were considered. Filtered reads were aligned to one-to-one transcripts using Bowtie2 (v. 2.2.9) [68] and RPKM values estimated using Cufflinks (v2.2.1) [69].

### 4.4. lncRNA Identification

The one-to-one transcripts were screened for lncRNA on several parameters. Transcripts longer than 200 bp with maximum open reading fame (ORF) size of less than 100 amino acids by Getorf (EMBOSS:6.6.0.0) were used to predict putative lncRNAs. These transcripts were analyzed using two software CPC (v.0.9-r2) [66] and PLEK (v1.2) [67] which also filter out transcripts by ORF size and number, transcript length and calculates the transcript coding capacity based on its features. Only transcripts classified as non-coding in both CPC and PLEK analysis were considered as putative lncRNA. Further we kept only those putative lncRNAs with RPKM ≥ 1.

### 4.5. Interspecies lncRNAs Conservation Analysis

To identify other lncRNAs which holds sequence conservation among Fabaceae species, we used BLASTN 2.7.1+ (e-value 10^−15^, identity 90% qcov 50%) [76] against the downloaded GREENC [122] and CANTATAdb v2.0 [63] libraries of Fabaceae lncRNAs, namely *Glycine max*, *Medicago truncatula*, *Phaseoulos vulgaris*; both databases present putative lncRNA obtained through their own bioinformatics pipeline, which is similar to the one we used to filter our own.

### 4.6. Interspecies lncRNA Genome Conservation Analysis

The copaíba putative lncRNA was then mapped through Bowtie2 (v2.3.4.1, default parameters) [68] to seven Fabaceae genomes available at NCBI Genomes (ftp://ftp.ncbi.nlm.nih.gov/genomes/): (i) *Vicia faba* (CSVX00000000), (ii) *G*. *max* (GCF_000004515.4.v2.0) [123], (iii) *M*. *truncatula* (GCA_000219495.2.v4.0) [71], (iv) *Phaseolus vulgaris* (GCF_000499845.1v1.0) [72], (v) *Lotus japonica* (GCA_000181115.2.v3.0) [73], (vi) *Vigna unguiculata* (GCA_001687525.1.v0.03) [74] and (vii) *Cicer reticulatum* (GCA_002896235.1.v0.03) [75]. The transcripts which aligned to the aforementioned genomes were compared to assess their conservation in the Fabaceae family using DrawVenn application (http://bioinformatics.psbugent.be/webtools/Venn/). It enabled the identification of lncRNAs which mapped to more than one genome.

### 4.7. Interspecies Conservation of Expressed lncRNA Analysis

The EST and cDNA from Fabaceae species were obtained from PlantGDB [124] and Phytozome [125], namely *C*. *reticulatum* [75], *P*. *vulgaris* [72], *M*. *truncatula* [71], *G*. *max* [122], *V*. *unguiculata* [73,74] and *L*. *japonicus* [73]. The copaíba lncRNA were compared to these Fabaceae transcripts using BLASTN 2.7.1+ (e-value 10^−15^, identity 90% qcov 50%) [76] followed by DrawVenn analysis (http://bioinformatics.psb.ugent.be/webtools/Venn/) to identify the ones which aligned to more than one species transcriptome. The lncRNA mapped in the genome and transcriptome analysis were selected accordingly to their expression for further analysis. 

### 4.8. Second Structure Modeling

The 1141 copaíba lncRNAs, which were regarded as conserved in the genome and transcriptome analysis, were selected for second structure modeling, using Vienna RNAfold (v2.4.8) [85] package, at 25 °C, default parameters. Subsequently the transcripts which presented second structure stability, evaluated through the MFE value, had their RPKM value compared between the two samples to investigate the potential regulation.

### 4.9. RT-qPCR Analysis of Copaíba lncRNA Expression

The total RNA was extracted using Japelaghi modified [91] protocol, the total RNA was treated with DNase followed by cDNA syntheses. The copaíba lncRNAs were selected due to their high conservation (transcriptome and genome analysis) and lower MFE value. These six candidates that had their expression analyzed were: JCF44_0000061237 (candidate 1), JCF44_0000046349 (candidate 2), JCF44_0000015840 (candidate 3), JCF44_0000094004 (candidate 4), JCF44_0000040403 (candidate 5) and JCF44_0000021616 (candidate 6). The RT-qPCR was performed using the standard protocol, the primers used are available at Table 2.

### 4.10. Computational Prediction of miRNA and lncRNA Interaction

The 1141 conserved copaíba lncRNA were analyzed in order to identify its possible role as miRNA targets or their involvement in the miRNA regulatory mechanisms as endogenous targets mimics. The 1960 mature miRNA sequences from nine Fabaceae species were obtained from miRBase (release 22) [87]. The miRNA target prediction was performed locally through psRobot [86] (default parameters, score < 4). The lncRNA–miRNA target mimicry prediction, was perfomed using psMimic with the default parameters as defined in Wu et al. [61], which considers that: (a) the 2nd to 8th positions at the 5′ end of a miRNA must be perfectly aligned to the target, (ii) three unpaired nucleotides are allowed between the 9th to 12th positions at the 5′ end of the miRNA sequence, and (iii) at most 3 nucleotide mismatch (excluding bulge region) can be between miRNA and lncRNA sequences. 

## Figures and Tables

**Table 1:** De novo transcript assembly and lncRNA identification pipeline results of copaíba from Atlantic Rain Forest (ARF) and Cerrado (CER).

Sample	Total Transcripts	Longest Contig per Cluster	CPC lncRNA Prediction	PLEK lncRNA Prediction	Overlapped lncRNA Predicted	One-to-One Correspondence (RPKM > 1)
ARF	138,175	94,815	67,251	86,608	64,801	8020
CER	199,556	140,011	102,804	129,710	99,570	8020

Abbreviations: lncRNA: long non-coding RNA; CPC: coding potential calculator; PLEK: predictor of long non-coding RNAs and messenger RNAs based on an improved k-mer scheme; RPKM: reads per kilobase of transcript per million mapped reads.

**Table 2:** The following primers were used to assess the relative expression of three selected lncRNAs which presented a higher degree of conservation and predicted second structure stability.

LncRNA ID	Forward Primer Sequence	Reverse Primer Sequence
Candidate 1	AATGCAATACAGCAACCTCTAAACC	GGAGGCACCTGGTGTATTGG
Candidate 2	TCATATCAATGCGGCACTCAA	TGTCTTCAGCTGCCCTTTCTG
Candidate 3	AGCAATTGCGGTTGGTATCC	TGGTACCTTTTCATGTTGCTTTCA
Candidate 4	TCAGGCAGCAGAGGAAGAATC	CACCCAGTTCATGCAACCAA
Candidate 5	CGCCAAATGTCCGCAGAT	GGACTTGCCCGCTATGCA
Candidate 6	AGCAATTGCGGTTGGTATCC	TGGTACCTTTTCATGTTGCTTTCA

## Supplementary Materials

Supplementary Materials are available online at http://www.mdpi.com/2311-553X/4/4/27/s1. Table S1: Presents the fasta sequence of the 8020 putative copaíba lncRNAs transcripts. Table S2: Presents the novel putative copaíba lncRNA identified to be regulated above 2-fold in the comparison of CER and ARF samples. In this table are the identification of the transcripts in each sample, separated by columns and its respective RPKM expression. Table S3: Presents the novel copaíba lncRNA which were expressed in both genome and transcriptome of other Fabaceae species at the conservation analysis. The table presents their identification in each sample, and the respective RPKM expression. Table S4: Presents novel copaíba lncRNA that were expressed in both genome and transcriptome of other Fabaceae species at the conservation analysis, and were regulated above 2-fold between CER and ARF samples, indicating they are potentially involved in the plants adaptive response to the different biomes. This table presents the identification in each sample, and the respective RPKM expression. Table S5: Presents the identification of a subset of conserved lncRNA, which underwent secondary structural prediction. This file features the RPKM and lncRNAs respective MFE predicted value. Table S6: Table presenting the lncRNA ID and the miRNA which is predicted to target it, provided by psRobot software. Table S7: Table presenting the identification of the lncRNA, which is predicted to act as a decoy target, and the miRNA associated with it. Figure S1: Copaíba lncRNA expression comparison analysis from RNA-Seq and RT-qPCR.
