# Supplementary material for: Copaifera langsdorffii Novel Putative Long Non-Coding RNAs: Interspecies Conservation Analysis in Adaptive Response to Different Biomes
Source: Noncoding RNA. 2018 Oct 8;4(4):27. doi: 10.3390/ncrna4040027 (PMC6316758; doi:10.3390/ncrna4040027)
Supplement: Supplementary file 1 [file ncrna-04-00027-s001.zip › Figure S1.doc]

(**A**)

(**B**)

**Figure S1.** Copaíba lncRNA expression comparison analysis from RNA-Seq and RT-qPCR. (**A**) RT-qPCR analysis of the relative RNA level expression of three candidate lncRNAs, * indicates that these replicates presented differential expression in comparison to ARF1 sample (p < 0.05). The ARF individuals presented a lower expression of the analyzed transcripts in comparison to CER individuals. CER-1 appears to have an enriched expression in comparison to its peers, which was not possible to evaluate through previous pool sequencing. Relative transcript levels expression was calculated using 28S gene expression as standard. (**B**) The RNA-Seq expression levels of the three candidates lncRNAs. In the CER population there is a higher expression of the given candidates 4 and 5, which agrees with the relative expression observed.
